# Effects of silicon application on leaf structure and physiological characteristics of *Glycyrrhiza uralensis* Fisch. and *Glycyrrhiza inflata* Bat. under salt treatment

**DOI:** 10.1186/s12870-022-03783-7

**Published:** 2022-08-04

**Authors:** Zihui Shen, Xiaojiao Cheng, Xiao Li, Xianya Deng, Xiuxiu Dong, Shaoming Wang, Xiaozhen Pu

**Affiliations:** 1 College of Life Sciences, Shihezi University, Shihezi, 832003 China; 2 Pharmacy School, Key Laboratory of Xinjiang Phytomedicine Resource and Utilization, Ministry of Education, Shihezi University, Shihezi, 832003 China

**Keywords:** Salinity, Foliar silicon application, Licorice, Anatomical structure, Photosynthesis

## Abstract

**Background:**

Soil salinization leads to a significant decline in crop yield and quality, including licorice, an important medicinal cash crop. Studies have proofed that the application of exogenous silicon can significantly improve the ability of licorice to resist salt stress, however, few studies concentrated on the effects of foliar silicon application on the morphology, physiological characteristics, and anatomical structure of licorice leaves under salt stress. In this study, the effects of Si (K_2_SiO_3_) on the structural and physiological characteristics of *Glycyrrhiza uralensis* Fisch. and *G. inflata* Bat. leaves under different salt concentrations (medium- and high-salt) were studied.

**Results:**

Compared with the control (without salt), the plant height, total dry weight, leaf area, leaf number, relative water content, xylem area, phloem area, ratio of palisade to spongy tissue, gas exchange parameters, and photosynthetic pigment content of both licorice varieties were significantly reduced under high-salt (12S) conditions. However, the thickness of the leaf, palisade tissue, and spongy tissue increased significantly. Applying Si to the leaf surface increased the area of the vascular bundle, xylem, and parenchyma of the leaf’s main vein, promoted water transportation, enhanced the relative leaf water content, and reduced the decomposition of photosynthetic pigments. These changes extended the area of photosynthesis and promoted the production and transportation of organic matter. *G. uralensis* had a better response to Si application than did *G. inflata*.

**Conclusions:**

In conclusion, foliar application of Si can improve water absorption, enhance photosynthesis, improve photosynthetic capacity and transpiration efficiency, promote growth and yield, and alleviate the adverse effects of salt stress on the leaf structure of the two kinds of licorice investigated.

**Supplementary Information:**

The online version contains supplementary material available at 10.1186/s12870-022-03783-7.

## Background

Presently, it is estimated that soil salinization affects one-fifth of total cultivated land and one-third of irrigated agricultural land [1]. Deterioration of the natural environment and inappropriate agricultural measures aggravate soil salinization every year, threatening the sustainable development of agricultural production and thereby resulting in considerable challenges for world agriculture [2]. Soil salinization is mainly characterized by high soil salt concentration, which has a significant negative impact on crop yield and quality, such as crop physiological drought, photosynthetic inhibition, nutrient imbalance, membrane system damage, and metabolic disorder [3–8], which seriously threaten crop growth and development and hinder the sustainable development of agriculture. High soil salt levels increase the absorption and accumulation of Na^+^, cause ion toxicity, reduce the membrane stability index and antioxidant enzyme activity of plants, and damage cell function [4]. Zhang et al. [9] observed that high salt concentrations could reduce leaf turgor and induce premature leaf senescence by disturbing the water relationship of crops. Arif et al. [10] showed that high salt concentrations obstruct photosynthesis and transpiration by destroying chloroplast structure, reducing photosynthetic pigment content, and closing stomata, resulting in greatly reduced crop yield. The loss of agricultural production caused by high-concentration salt areas worldwide exceeds $12 billion every year [11]. The development and utilization of a large area of salinized land and improving salt resistance and tolerance of crops has thus become a current research focus.

*Glycyrrhiza uralensis* Fisch., and *G. inflata* Bat. are two original plants of licorice, mainly distributed in the saline areas of northwest China [12]. They have various pharmacological effects and exhibit cold resistance, drought resistance, and salinity resistance due to their well-developed underground roots and rhizomes and high ground coverage. Therefore, they are important plant resources for saline-alkaline land improvement in arid and semi-arid areas of Northwest China [9]. In recent years, the market demand for licorice resources has been increasing because of the improvement in the licorice medicinal value and the expansion of its fields of application. Wild licorice resources are very scarce and are on the verge of depletion due to long-term overexploitation [13]. As such, artificial cultivation of licorice is an effective measure to realize sustainable utilization of licorice resources. However, soil salinization has become increasingly prominent with the deterioration of the ecological environment. Studies have shown that with an increase in environmental salt concentration (≥ 6 g kg^–1^ NaCl), the growth, development, and quality of licorice are inhibited to varying degrees [9, 13], ultimately leading to a decrease in its medicinal and economic value.

The leaf is an important photosynthetic organ, and its anatomical characteristics are key to determining the survival ability of plants in a specific environment [14]. Chlorophyll (Chl) content and gas exchange parameters in leaves are important indicators of plant physiological characteristics and serve as important factors affecting photosynthetic intensity [15, 16]. Arif et al. [10] showed that salinity reduced the Chl and carotenoid (Car) content, resulting in a decrease in the net photosynthetic rate and stomatal conductance, which hindered photosynthesis and significantly reduced crop biomass. The normal structure is the basis of its physiological function; a change in the former correspondingly leads to a change in its function. For example, changes in the structure of xylem vessels directly lead to changes in hydraulic conductivity [17], thus affecting its ability to absorb water. In addition, studies showed that high salt concentrations led to decreased leaf area and number, increased thickness of leaves and epidermis, reduced the number and diameter of xylem vessels, enhanced palisade parenchyma volume and intercellular space, and decreased spongy parenchyma volume [18, 19], showing leaf structure destruction and the weakening of photosynthetic capacity. The leaf morphology, physiological characteristics, and anatomical structure of plants are gradually formed by adapting to the environment for a long time during the evolution process, which reflects the adaptive strategy of plants to the environment [20]. However, changes in morphology, structure, or physiology alone cannot adequately simulate leaf senescence or death when the system is stressed, directly impacting plant productivity. With the increase in mortality, leaf morphology or function changes may improve the plants’ sensitivity to stress and even subsequent disorder [21].

Silicon (Si) is an environmentally friendly element that does not cause corrosion or pollution [22]. It can accumulate in the cell walls of plants in the form of SiO_2_ to enhance the mechanical effects of the cell wall. Concurrently, it can also be used as a physical barrier to reduce water loss in plants and improve the ability of plants to resist environmental stress [3, 23]. Si plays an important role in improving photosynthesis and ion homeostasis, enhances antioxidant defense capacity, and reduces lipid peroxidation and electrolyte leakage [4–8, 24, 25]. Hurtado et al. [5] studied the antioxidant defense mechanism of sorghum and sunflower under NaCl stress through different Si application methods. They found that, compared with the combination of root and leaf-root applications, foliar application of Si reduced Na^+^ absorption and lipid peroxidation, improved antioxidant enzyme activity, leaf relative water content, leaf area, and aboveground biomass, and alleviated the damage caused by high salt. Laane [22] also concluded that foliar Si application had a more significant beneficial effect on plants. When Si is directly applied to the soil, many variables, from the soil–water environment to the water balance of plants, may affect the absorption and transportation of Si by plant roots. However, its foliar application can ensure that silicic acid can be directly applied to plants more accurately and that the growth-promotion effects are more significant [22, 26]. How the foliar application of Si affects the morphological, physiological, and structural characteristics of licorice leaves, and the nature of the relationships among them remains unknown. However, it is important to understand the mechanism of the effect of Si on the important physiological functions of licorice and fully tap the biological potential of licorice leaves. Therefore, this study used foliar application of Si to reveal its effects on *G. uralensis* and *G. inflata* leaves under different salt concentrations. We hypothesized that Si application could significantly improve the internal structure of leaves under salt treatment, adjust leaf morphological characteristics, promote gas exchange parameters, increase photosynthetic pigment content, enhance the photosynthetic process, thus ameliorating the effects of saline stress.

## Results

### Effects of Si and NaCl treatment on leaf morphological characteristics

**Fig. 1:**
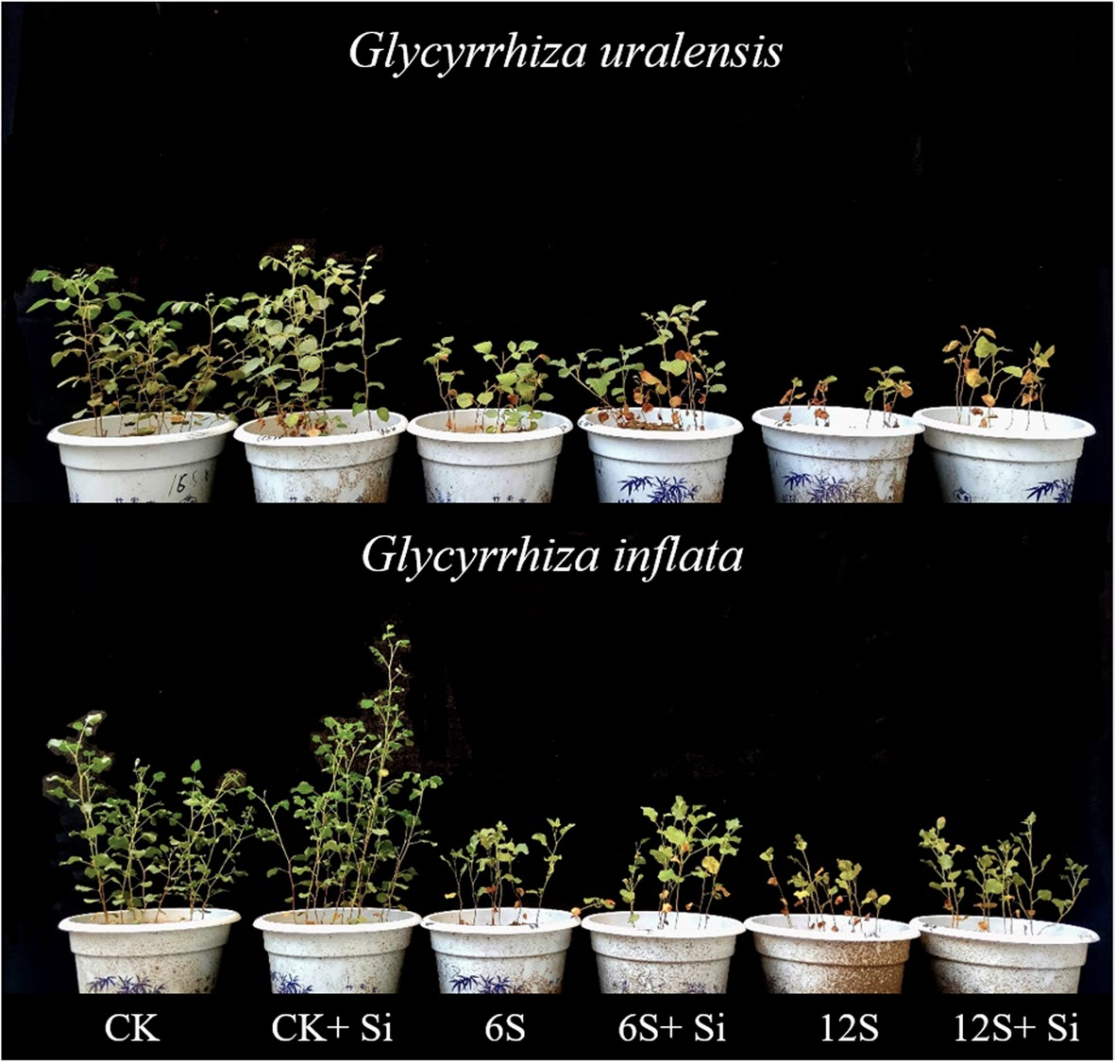
Effects of Si and NaCl treatment on the morphology of two varieties of licorice

**Fig. 2:**
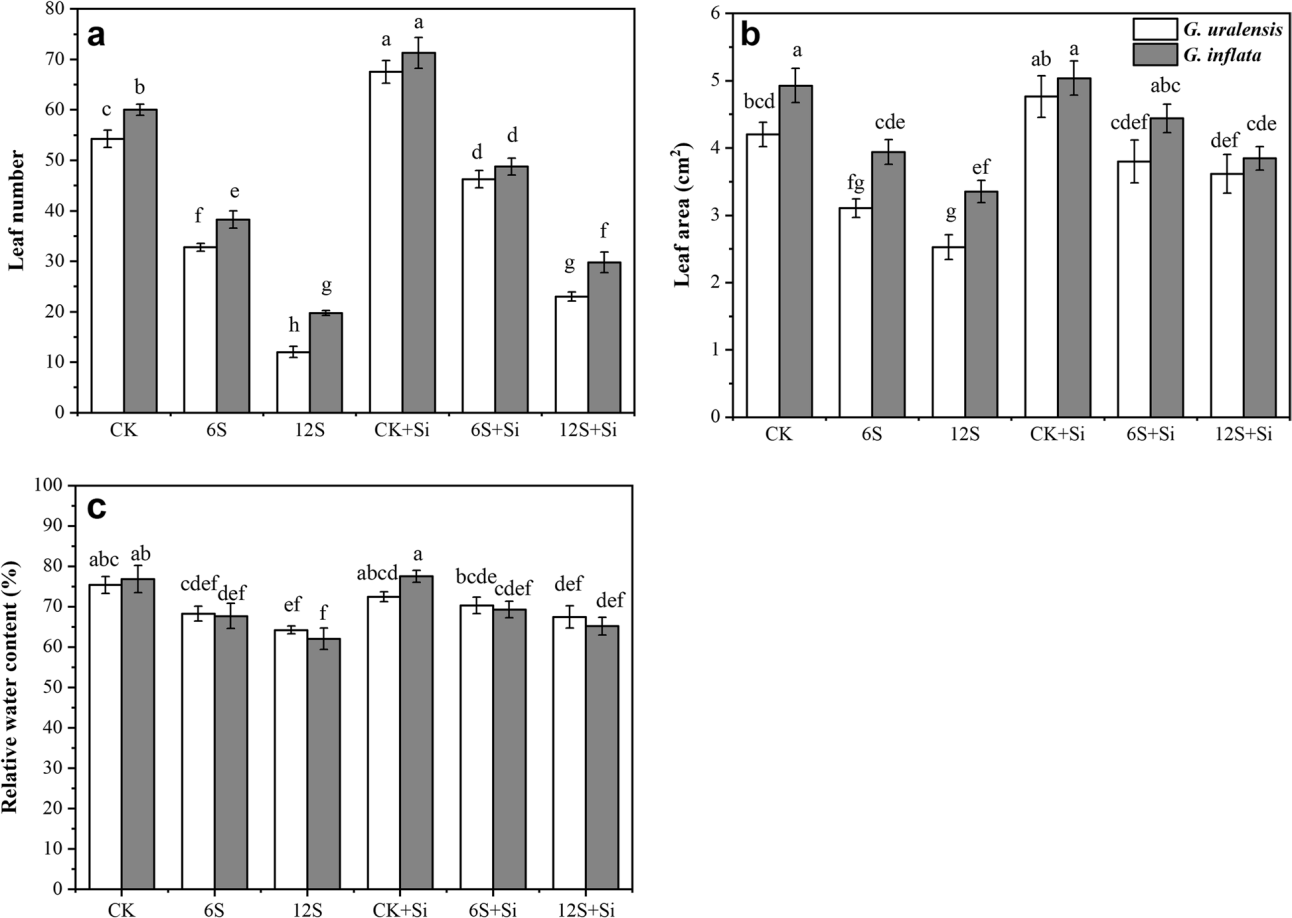
Effects of Si and NaCl treatment on leaf number (**a**), leaf area (**b**), and relative water content (**c**) of two varieties of licorice. Results are mean for four replicates with different letters indicating differences at *P* < 0.05 by Duncan’s multiple comparison tests

There were significant effects of Si (*P* < 0.01) and NaCl treatment (*P* < 0.01) on the leaf area and number of leave of two varieties of licorice. The NaCl treatment also significantly affected relative water content of two varieties of licorice (*P* < 0.01). However, the interaction between Si and NaCl treatment had no significant effect on leaf area, leaf number and relative water content of two varieties of licorice (Table S1). With an increase in salt concentration, the number of leaves, leaf area, and relative water content of the two licorice varieties decreased significantly compared with those of CK, while the addition of Si alleviated this inhibition (*P* < 0.05) (Figs. 1 and 2). The supply of Si under 12S stress increased the leaf number and leaf area of *G. uralensis* by 92 and 43%, respectively, and that of *G. inflata* by 51 and 15%, respectively, compared to the plants grown under 12S stress alone. In addition, compared to plants lacking Si application (CK, 6S, and 12S), foliar Si application (CK + Si, 6S + Si, and 12S + Si) increased the relative water content of the leaves of both licorice varieties.

### Effects of Si and NaCl treatment on leaf anatomical structure

**Fig. 3:**
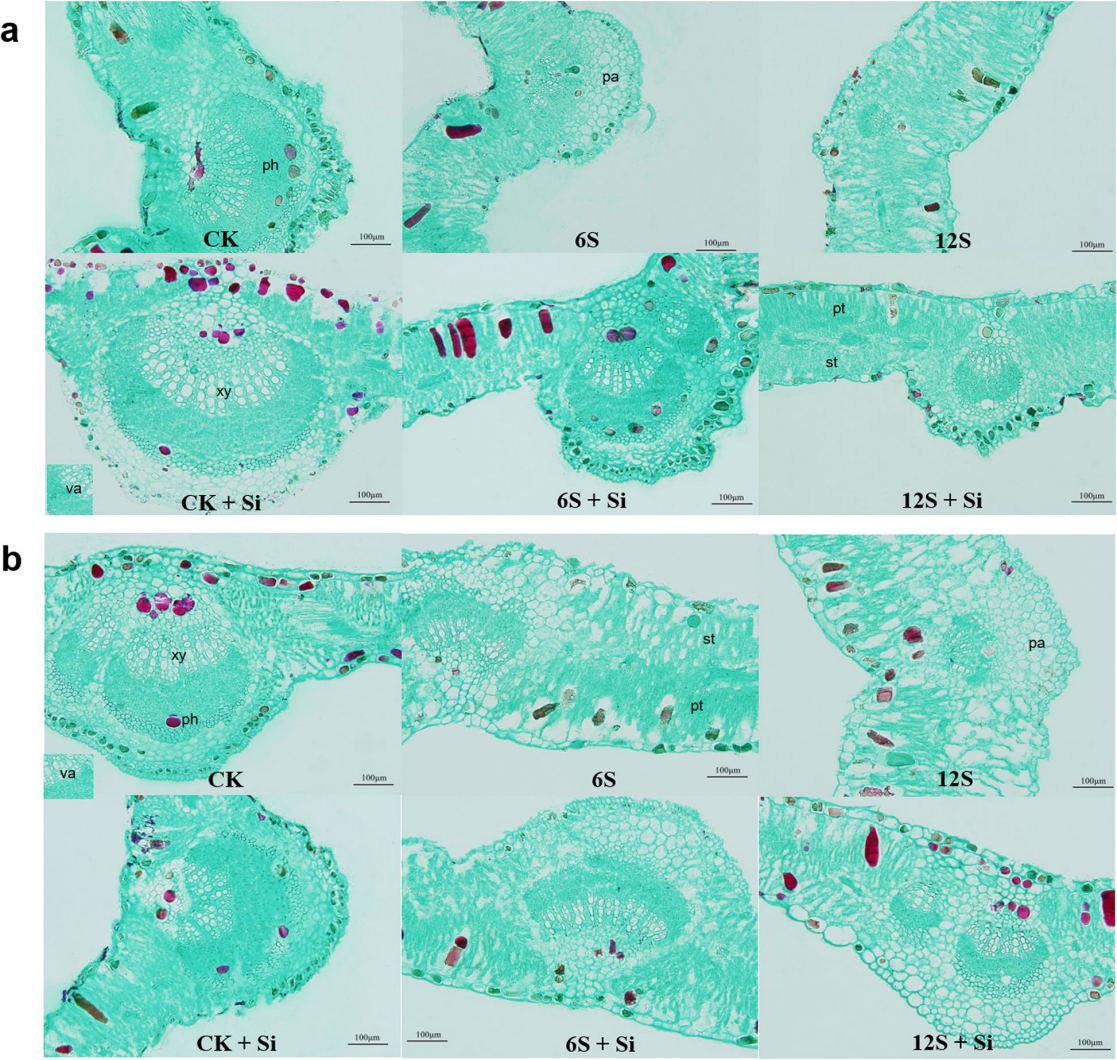
Effects of Si and NaCl treatment on leaf anatomical structure of *Glycyrrhiza uralensis* (**a**) and *G. inflata* (**b**). co: collenchyma, xy: xylem, ph: phloem, va: vascular bundle, pa: parenchyma, pt: palisade tissue, st: spongy tissue. Scale bar: 100 µm

With increased salt concentration, the leaf thickness and mesophyll cell gap of the two varieties of licorice gradually increased, the cell arrangement loosened, and the vascular bundle area of the main vein shrank (Fig. 3). Under 12S treatment, the mesophyll cells of the two varieties of licorice appeared to rupture. After spraying Si on the leaf surface, the cell gaps in the mesophyll of the two licorice varieties gradually narrowed, the arrangement between cells became closer, and the area of the vascular bundles of the main veins increased.

**Fig. 4:**
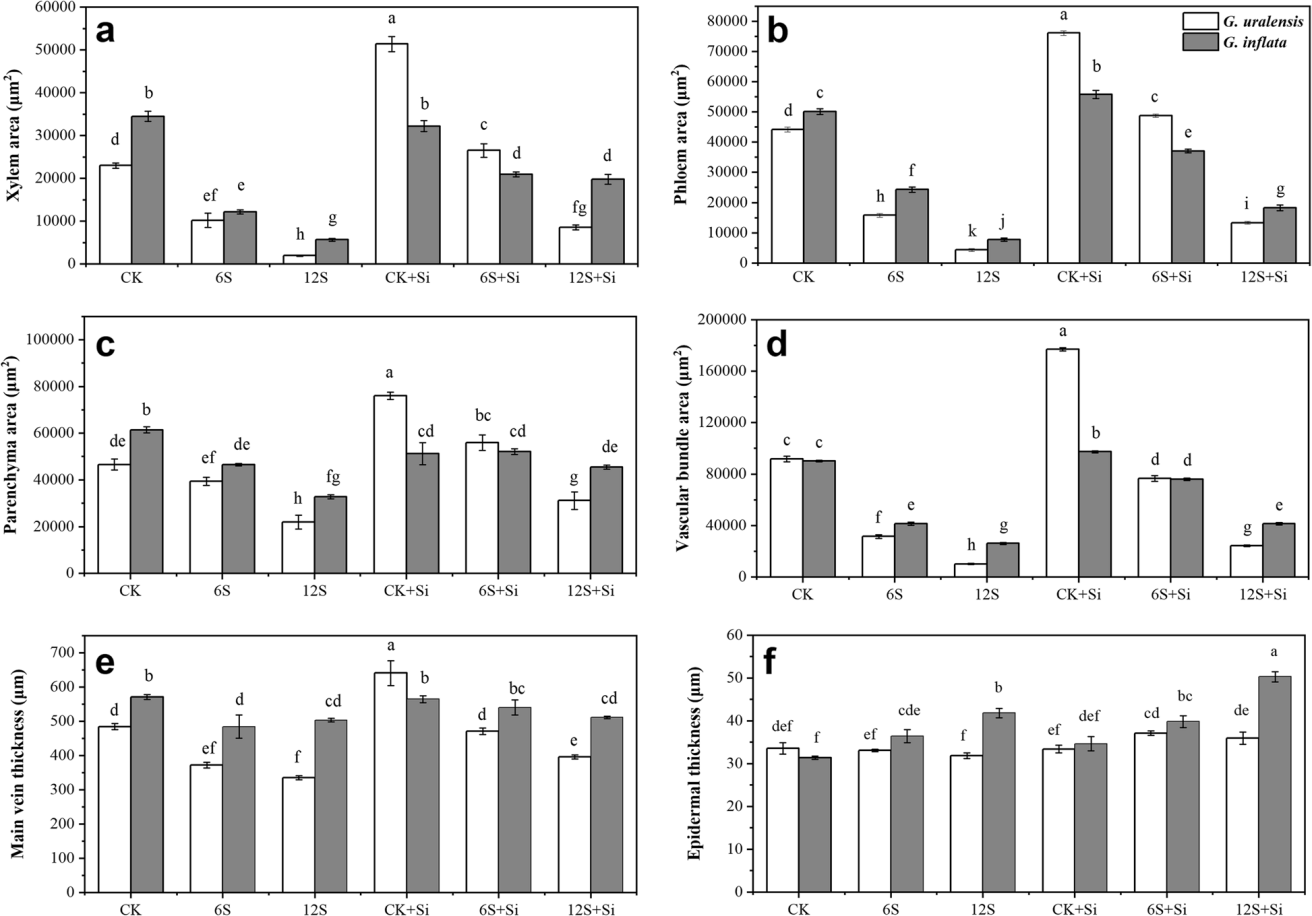
Effects of Si and NaCl treatment on xylem area (**a**), phloem area (**b**), parenchyma area (**c**), vascular bundle area (**d**), main vein thickness (**e**), and epidermal thickness (**f**) of two varieties of licorice. Results are mean for four replicates with different letters indicating differences at *P* < 0.05 by Duncan’s multiple comparison tests

**Fig. 5:**
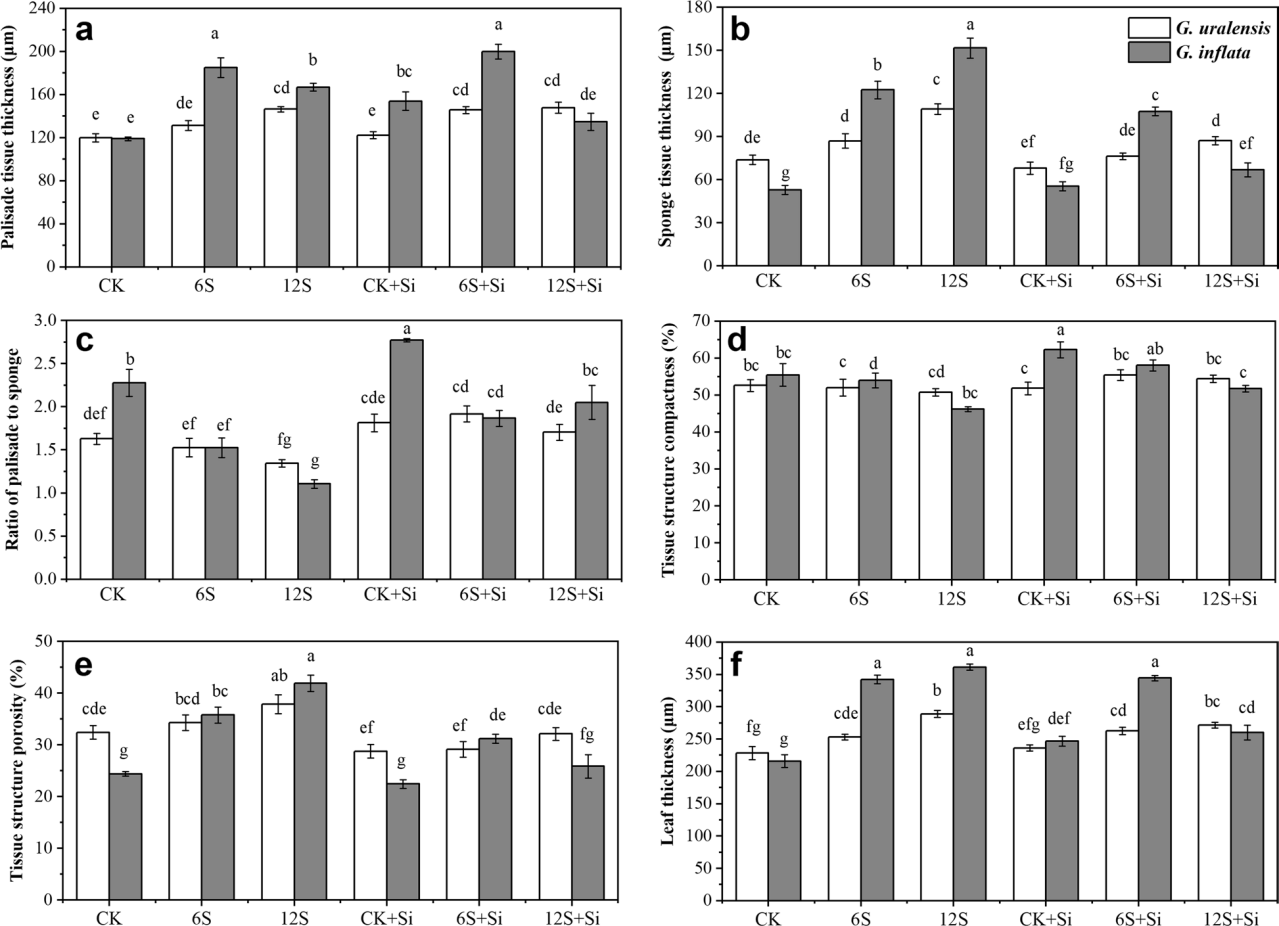
Effects of Si and NaCl treatment on palisade tissue thickness (**a**), spongy tissue thickness (**b**), the ratio of palisade to spongy (**c**), tissue structure compactness (**d**), tissue structure porosity (**e**), and leaf thickness (**f**) of two varieties of licorice. Results are mean for four replicates with different letters indicating differences at *P* < 0.05 by Duncan’s multiple comparison tests

There were significant effects of Si (*P* < 0.01), NaCl treatment (*P* < 0.01) and their interactions (*P* < 0.01) on the xylem area, phloem area and vascular bundle area of two varieties of licorice (Table S1). According to Fig. 4, the xylem, phloem, parenchyma, vascular bundle, and main vein thicknesses of *G. uralensis* were significantly reduced under the 12S treatment, and the level of reduction (91, 90, 53, 89, and 31%) were greater than those of *G. inflata* (83, 85, 47, 71, and 12%, respectively). In contrast to *G. uralensis*, the epidermal thickness of *G. inflata* increased significantly by 16 and 33% under the 6S and 12S treatments, respectively. Compared with CK, the leaf thickness, palisade tissue thickness, spongy tissue thickness, and tissue structure porosity of the two varieties of licorice under the 12S treatment increased significantly, with a greater increase observed in *G. inflata* (Fig. 5). Therefore, the xylem, phloem, and vascular bundle areas of the main vein of *G. uralensis* under 12S + Si treatment increased by 333, 204, and 138%, respectively, and the corresponding increases in *G. inflata* were 246, 136, and 59%, respectively, compared to the 12S treatment. Compared with no Si treatment (CK, 6S, and 12S), Si application (CK + Si, 6S + Si, and 12S + Si) increased the epidermal thickness and tissue structure compactness of the two licorice varieties but reduced the spongy tissue thickness and tissue structure porosity. For *G. inflata*, the 12S + Si treatment reduced the leaf thickness (28%), the palisade thickness and spongy tissues simultaneously, with a greater decrease in spongy tissue (55%), compared to the 12S treatment; thus, the ratio of palisade to spongy tissue increased (Fig. 5c).

### Effects of Si and NaCl treatment on leaf photosynthetic pigments

**Fig. 6:**
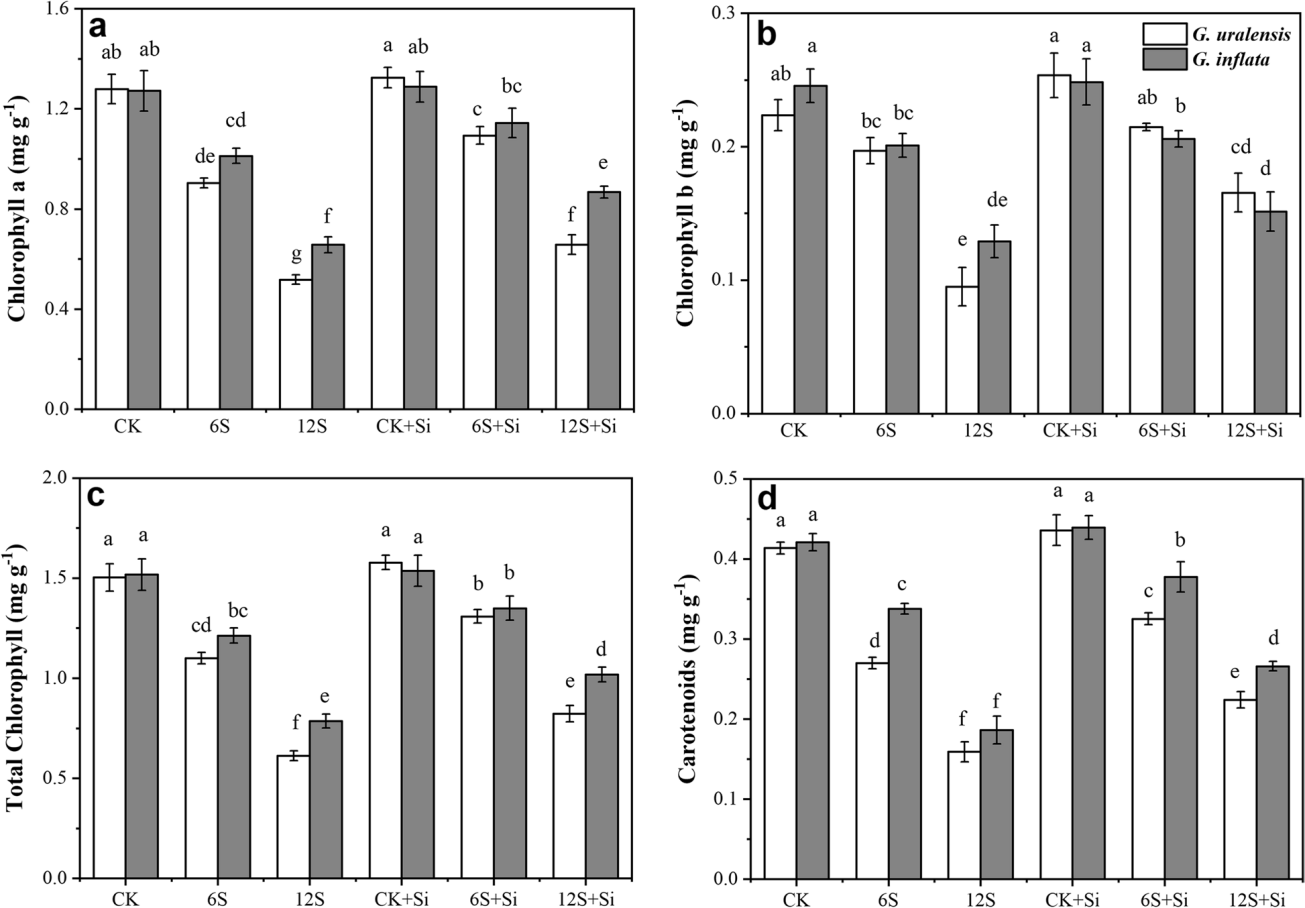
Effects of Si and NaCl treatment on chlorophyll a (**a**), chlorophyll b (**b**), total chlorophyll (**c**), and carotenoid content (**d**) of two varieties of licorice. Results are mean for four replicates with different letters indicating differences at *P* < 0.05 by Duncan’s multiple comparison tests

There were significant effects of Si (*P* < 0.01), NaCl treatment (*P* < 0.01) and their interactions (*P* < 0.01) on the Chl a, total Chl, and Car contents of two varieties of licorice, but the interaction between Si and NaCl treatment had no significant effect on the Chl a, total Chl, and Car contents of two varieties of licorice (Table S1). As shown in Fig. 6, NaCl treatment significantly reduced the content of all photosynthetic pigments in both licorices. Under the 12S treatment, the Chl a, Chl b, total Chl, and Car contents of *G. uralensis* were significantly reduced, and the level of reduction (60, 57, 59, and 62%) were higher than those in *G. inflata* (48%, 47%, 48%, 56%). Application of the Si 12S treatment resulted in higher contents of Chl a (increased by 27 and 32%), Chl b (increased by 74 and 17%), total Chl (increased by 34 and 30%), and Car (increased by 41 and 43%, respectively), compared to the 6S + Si treatment.

### Effects of Si and NaCl treatment on leaf photosynthetic gas exchange parameters

**Fig. 7:**
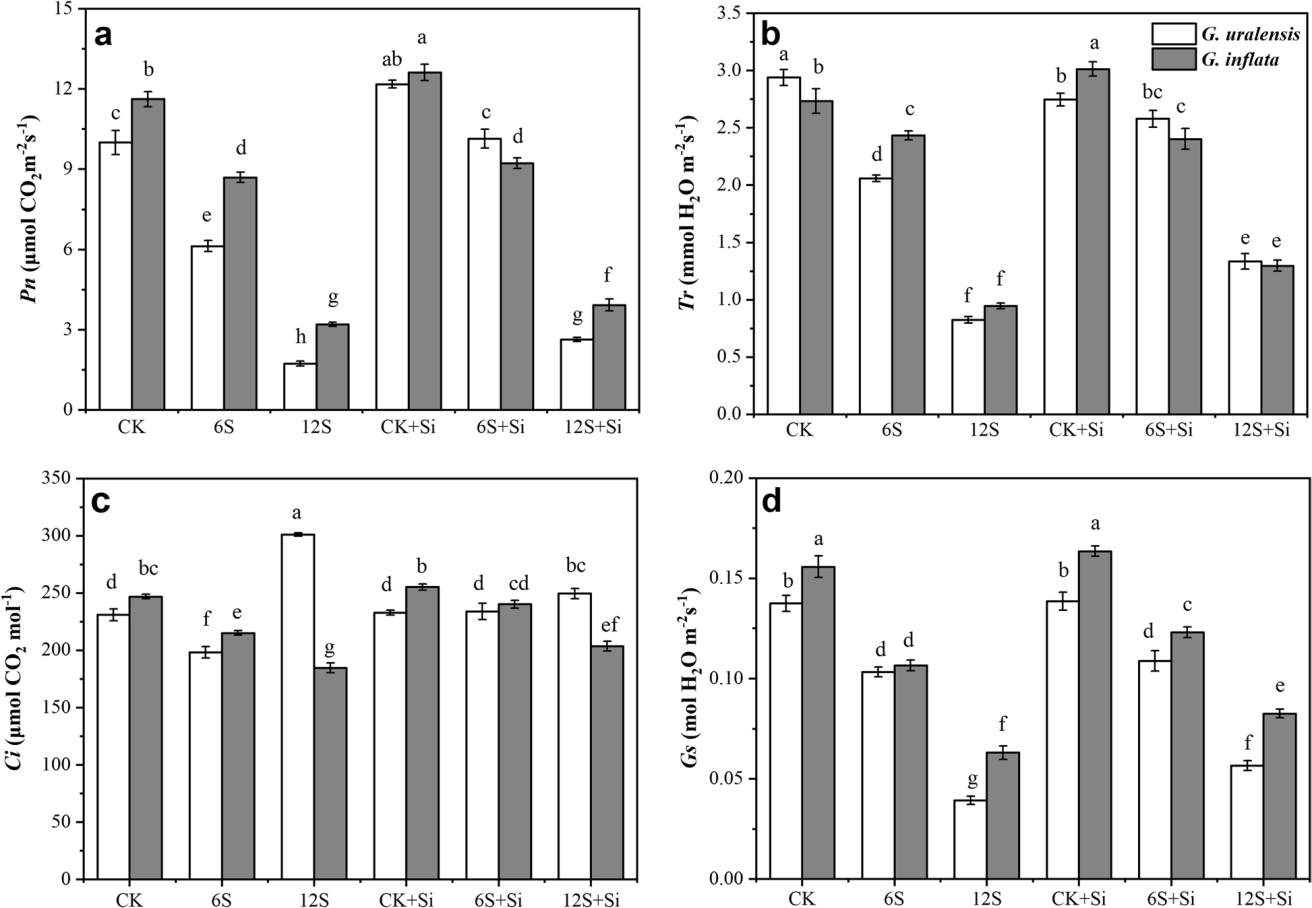
Effects of Si and NaCl treatment on net photosynthetic rate (*Pn*, **a**), transpiration rate (*Tr*, **b**), intercellular CO_2_ concentration (*Ci*, **c**), and stomatal conductance (*Gs*, **d**) of two varieties of licorice. Results are mean for four replicates with different letters indicating differences at *P* < 0.05 by Duncan’s multiple comparison tests

There were significant effects of Si (*P* < 0.01) and NaCl treatment (*P* < 0.01) on *Pn*, *Tr*, *Ci*, and *Gs* of two varieties of licorice, expect for *Ci* of *G. uralensis*. The interaction between Si and NaCl treatment also had significant effect on *Tr* and *Ci* of two varieties of licorice, but had no significant effect on *Gs* (Table S1). Compared with CK, 6S treatment significantly reduced the *Pn*, *Tr*, *Ci*, and *Gs* of both licorice varieties, whereas 12S treatment significantly increased the *Ci* of *G. uralensis* (30%) (Fig. 7). Conversely, foliar Si application in the 12S treatment significantly increased (*Pn*) (53 and 23%), *Tr* (62 and 37%), and *Gs* (44 and 31%) of *G. uralensis* and *G. inflata*, respectively, and significantly reduced the *Ci* (17%) of *G. uralensis*, compared to the 12S treatment.

### Effects of Si and NaCl Treatment on licorice growth parameters

**Fig. 8:**
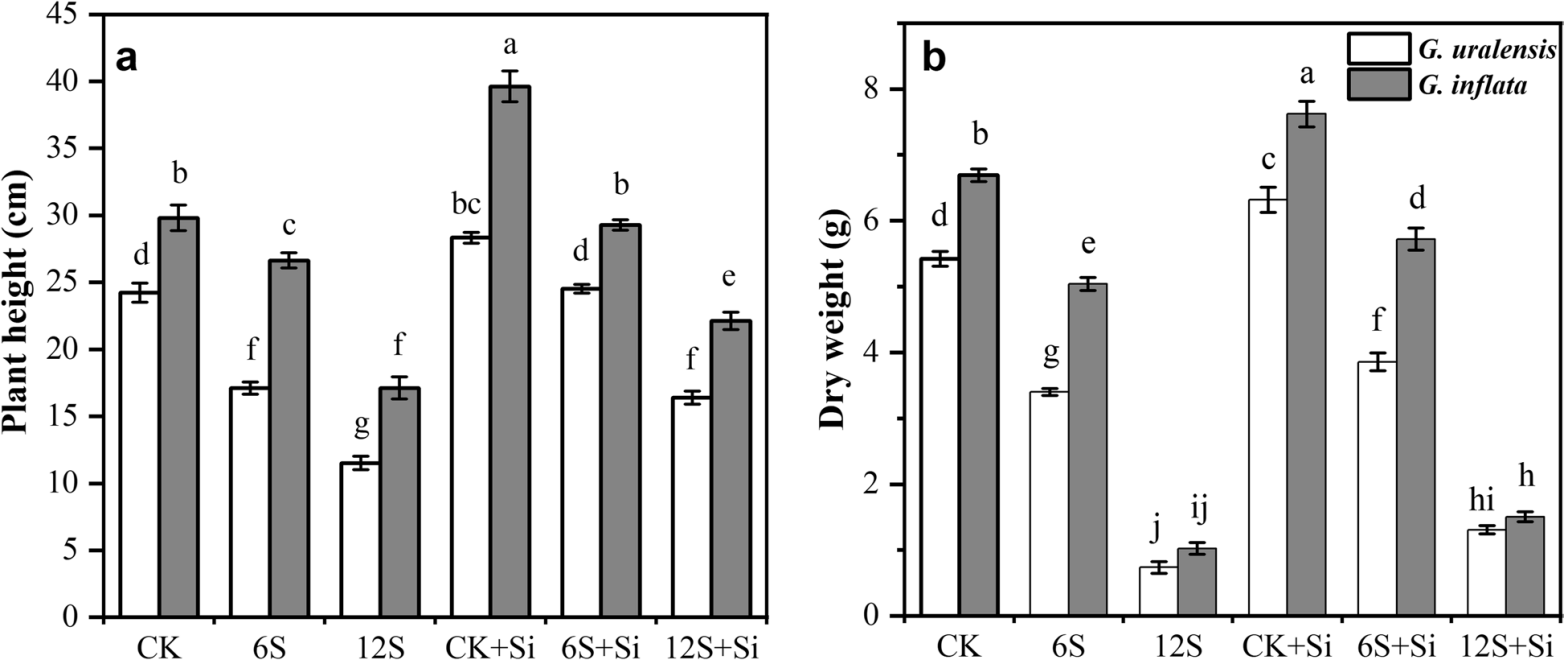
Effects of Si and NaCl treatment on plant height (**a**) and dry weight (**b**) of two varieties of licorice. Results are mean for four replicates with different letters indicating differences at *P* < 0.05 by Duncan’s multiple comparison tests

There were significant effects of Si (*P* < 0.01), NaCl treatment (*P* < 0.01) and their interactions (*P* < 0.05) on the plant height of two varieties of licorice. The NaCl treatment also significantly affected the dry weight of two varieties of licorice (*P* < 0.01), but the interaction between Si and NaCl treatment had no significant effect on the dry weight of two varieties of licorice (Table S1). Salt treatment significantly reduced the plant height and dry weight of the two licorice varieties (Figs. 1 and 8). In particular, under the 12S treatment, compared with CK, the plant height of *G. uralensis* and *G. inflata* decreased by 52 and 43%, respectively, and the dry weight decreased by 86 and 85%, respectively. In contrast, after foliar application of Si, the plant height and dry weight of the two licorice varieties increased significantly. The application of Si under the 12S treatment increased plant height and dry weight by 42 and 78%, respectively, in *G. uralensis*, and by 29 and 47% in *G. inflata*, respectively, compared with the 6S + Si treatment.

### Correlation analysis

**Fig. 9:**
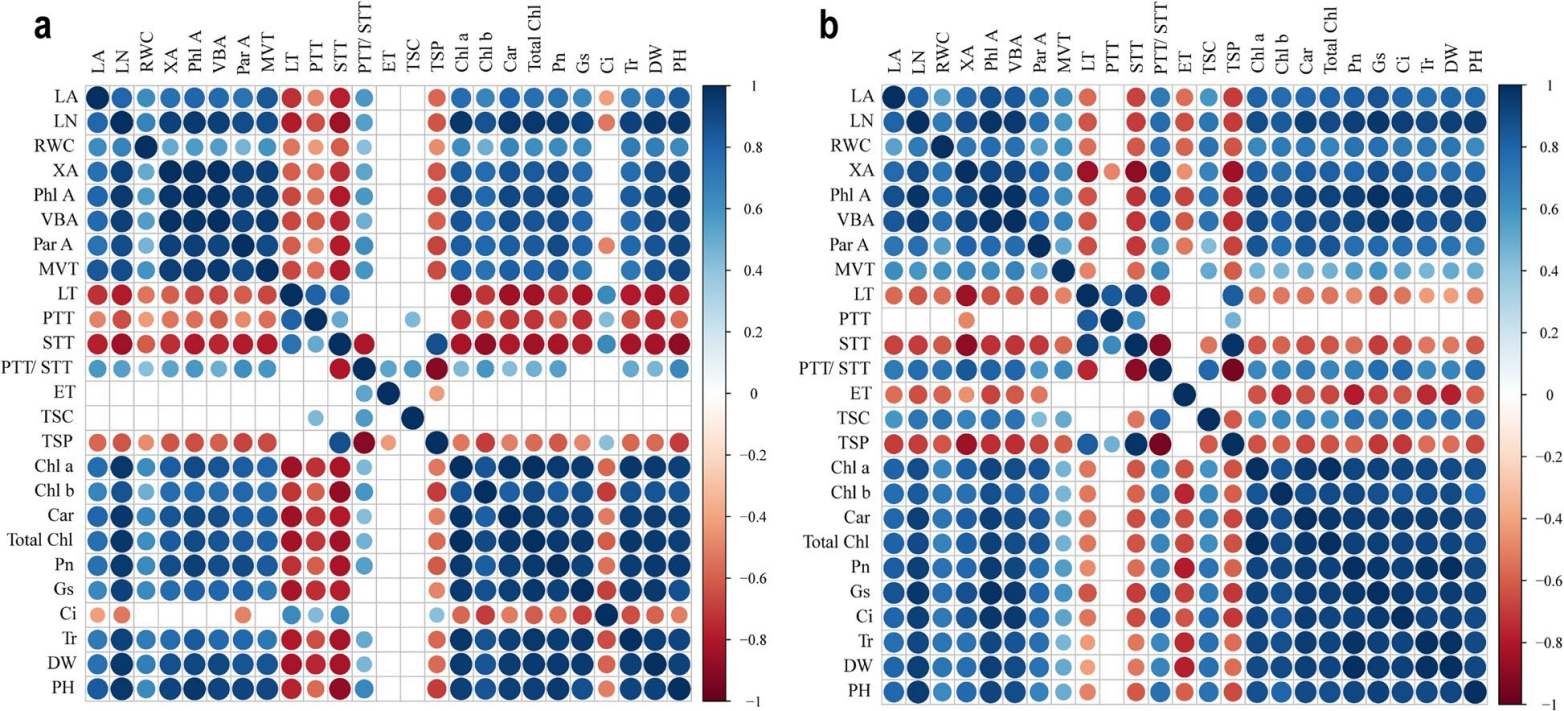
Correlation analysis (*P* < 0.05) between various measured attributes of *Glycyrrhiza uralensis* (**a**) and *G. inflata* (**b**). The abbreviations are as follows: LA (Leaf area), LN (Leaf number), RWC (Relative water content), XA (Xylem area), Phl A (Phloem area), VBA (Vascular bundle area), Par A (Parenchyma area), MVT (Main vein thickness), LT (Leaf thickness), PTT (Palisade tissue thickness), STT (Sponge tissue thickness), PTT/ STT (Ratio of palisade to sponge), ET (Epidermal thickness), TSC (Tissue structure compactness), TSP (Tissue structure porosity), Chl a (Chlorophyll a), Chl b (Chlorophyll b), Tocal Chl (Total Chlorophyll), Car (Carotenoid), *Pn* (Net photosynthesis rate), *Tr* (Transpiration rate), *Gs* (Stomatal conductance), *Ci* (Internal CO_2_ concentration), DW (Dry weigh), PH (Plant height)

Pearson’s correlation analysis was performed to monitor the differences on the morphology, physiological characteristics, and anatomical structure of *G. uralensis* and *G. inflata* leaves (Fig. 9). The dry weight of two varieties of licorices showed a positive correlation with leaf morphological characteristics (leaf area, leaf number and relative water content), the area of xylem, phloem, parenchyma and vascular bundle, main vein thickness, photosynthetic pigments, photosynthetic gas exchange parameters (except for *Ci* of *G. uralensis*) and plant height, and showed a negative correlation with leaf thickness, sponge tissue thickness and tissue structure porosity. Meanwhile, the gas exchange parameters and photosynthetic pigment content of the two varieties of licorices were positively correlated with the area of xylem, phloem, parenchyma and vascular bundle, main vein thickness and ratio of palisade tissue to sponge tissue, and negatively correlated with leaf thickness and sponge tissue thickness.

## Discussion

The response of plants to salt depends on the plant species and the time of exposure to the salt environment, the salt concentration, and salt type [27, 28]. Similarly, the effects of Si application are also different because of the different plant species and salinity [4, 29]. In this study, both medium and high salt concentrations inhibited the morphological characteristics, anatomical structure, gas exchange parameters, photosynthetic pigment content, plant height, and dry weight of *G. uralensis* and *G. inflata* leaves. Foliar application of Si to the leaves alleviated the inhibitory effect of salinity on the leaves of the two types of licorice, but the mitigating effect varied with the licorice variety.

Generally, plants undergo morphological and structural changes under high salt concentrations, in which the reduction in leaf area and number is regarded as two typical responses [30, 31]. High salt concentrations in the soil reduce the water potential of the rhizosphere environment and cause water stress in plants; the relative water content of plants is thus regarded as an important index of the salt tolerance mechanism [32]. The reduction of leaf area and number under high salt concentration limited water loss caused by transpiration but reduced photosynthetic area to a certain extent, thus decreasing the aboveground productivity of the plants [30]. In the present study, foliar application of Si effectively increased the leaf area, leaf number, and relative water content of *G. uralensis* and *G. inflata* under the two NaCl treatments, similar to Hurtado et al. [5] in sorghum and sunflower. After applying Si to the leaf surface, Si was absorbed by the leaves into the inner leaf tissue and deposited in the cell wall [9]. Due to the hydrophilic characteristics of Si, the treatment promoted water accumulation in the leaves, increased the water potential difference between leaves and roots, improved the water pull, enhanced the absorption of soil water and nutrients by roots, and promoted the growth and development of aboveground leaves. This coordinated leaf-root development further promoted the growth of the whole plant and effectively alleviated the toxic effects of Na^+^ and Cl^−^ on the plants [33]. In addition, some studies have pointed out that Si promotes the opening of leaf stomata by increasing the relative water content of leaves, conducive to the photosynthetic rate and plant growth [9, 32, 34].

Structural changes in the leaves directly affect the physiological and ecological functions of plants. Therefore, understanding the response of leaf structure to the environment is an important basis for exploring the mechanisms of plant adaptation [35]. The xylem transports water and inorganic nutrients for photosynthesis, whereas the phloem transports photosynthetic products and signaling molecules from the mesophyll to other parts of the plant [36]. For dicotyledonous plants, the well-developed vascular bundle structure of the main vein can ensure the transportation of water and nutrients, whereas the parenchyma in the main vein is responsible for storage [9]. In this study, the xylem, phloem, and vascular bundle areas in the main vein of the two licorice leaves were significantly reduced under salt treatment, and this adverse effect was more obvious in *G. uralensis*. Sarker et al. [11] showed that salt treatment reduces the diameter of the catheter cavity, resulting in a reduction of xylem area, thus reducing salt intake, conducive to better adaptation of plants to the salt environment. In addition, the reduction in the cross-sectional area of vascular bundles, especially xylem vessels, sharply weakens the conduction capacity of xylem and phloem, which reduces the transportation of Na^+^ in the soil environment by roots and the water transportation from roots to aboveground parts, and then decreases the accumulation of Na^+^ in tissues [37]. After Si application on the leaf surface, the area of xylem, phloem, vascular bundle, and parenchyma in the main vein of leaves of both licorice varieties increased significantly despite salt stress. The leaf vein thickness also increased, which improved the water transmission and storage capacity of the plant to a certain extent, diluted the concentration of salt ions in the tissue, and promoted the transportation of nutrients and photosynthetic products; these changes are known to ameliorate the adverse effects of salt on plants [38]. In addition, Si deposition on the leaf cell wall increases the affinity of xylem vessels for water and reduces water loss, thus increasing the relative water content of plants [39], which was also reflected in the results of this study.

The mesophyll is the main component of leaf photosynthesis. In the mesophyll parenchyma, the difference in thickness between palisade and spongy tissues directly affects the distribution of chloroplasts and the efficiency of photosynthesis [31]. In this study, the thickness of the leaves, main vein, palisade tissue, and spongy tissue, and porosity of the tissue structure porosity of the two varieties of licorice increased under salt treatment. In contrast, the ratio of palisade to spongy and tissue structure compactness decreased, similar to the results for *Lycium ruthenicum* [20] and zucchini squash [19]. After Si foliar application under medium salt treatment, these indices changed in the opposite way, similar to Taha et al. [38] on wheat. The ratio of palisade to spongy tissue of the leaves of the two licorices varieties can effectively improve the diffusion of CO_2_ in the mesophyll after applying Si under medium salt treatment, thereby compensating for stomatal limitation caused by the salt environment [27]. Concurrently, the increase in palisade tissue thickness and decrease in spongy tissue thickness can help CO_2_ effectively diffuse to chloroplasts under reduced stomatal aperture, ensuring the normal photosynthetic reaction [18, 27, 31]. In addition, an increase in the thickness of the palisade tissue further expands the surface area of the mesophyll, increases the number of chloroplasts, increases the chlorophyll content, enhances the ability of leaves to capture light energy, and promotes photosynthesis [31]. Under high-salt treatment with Si application, leaf thickness, especially of palisade and spongy tissues, was significantly reduced (Fig. 5a, b and f). This may be due to thinner leaves being able to bring mesophyll cells closer to the epidermis, which not only reduces the diffusion distance of CO_2_ from the surrounding environment to chloroplasts but also reduces the distance required for light to radiate through the leaves [34]. In addition, although the decrease in the thickness of palisade and spongy tissues reduced the area of mesophyll, *Ci,* and *Pn* in leaves increased significantly in this treatment, which showed that the main factor affecting photosynthesis under high-salt treatment was CO_2_ concentration, rather than the area of photosynthetic reaction.

Chlorophyll is important for capturing light energy during photosynthesis. Therefore, its content level is an important indicator of plant physiological characteristics and the basis of photosynthesis [15]. This study found that the content levels of photosynthetic pigments in the leaves of two varieties of licorice decreased significantly under salt treatment, which typically leads to a “short supply” of chlorophyll and hinders the photosynthetic reaction [15, 24]. This may be due to the inhibition of chlorophyll enzyme synthesis under high salt concentrations, which hindered the process of chlorophyll synthesis [40] or because the high salt levels inhibited the synthesis of ribulose-1,5-bisphosphate carboxylase [24], leading to the degradation of chloroplast structure and a decrease in chlorophyll concentration. However, Si foliar application to leaves effectively alleviated the adverse effects of salt treatment on the photosynthetic pigments of the two licorices varieties. Furthermore, it has been reported that Si in leaves can increase the levels of chlorophyll precursors (protoporphyrin IX, Mg-protoporphyrin IX, and protochlorophyllide), and expressions of genes encoding enzymes (*CHLH*, *POR,* and *CAO*) in chlorophyll synthesis, thus increasing the synthesis of chlorophyll [6], promoting the ability of leaves to capture light energy and enhancing the *Pn* [15]. In addition, Si can alleviate damage to the ultrastructure of chloroplasts caused by salt ions, increase the number of chloroplasts, and improve the chlorophyll content [9].

In this study, medium-salt treatment inhibited the *Pn*, *Tr*, *Ci*, and *Gs* of the two licorice varieties, consequently resulting in limiting photosynthesis by stomatal factors, consistent with the results of Zhang et al. [9]. In contrast, under high-salt treatment, the *Pn* of *G. uralensis* leaves decreased significantly, and the *Ci* increased significantly, probably due to high salt significantly reducing the *Gs* of the leaves (Fig. 7d), thus affecting the diffusion of CO_2_ inside and outside the leaves. This reduced the carbon assimilation capacity of the leaves, resulting in a large amount of CO_2_ which could not be used for carbohydrate synthesis, instead remaining in the intercellular space, where photosynthesis was limited by non-stomatal factors [41]. In contrast, Si foliar application increased the photosynthetic parameters of the two varieties of licorice. The reasons may be that (1) to a certain extent, Si can maintain the structural integrity of mesophyll cells and chloroplasts, ensure the number of chloroplasts, and increase the chlorophyll content [9]; (2) Si application on the leaf surface reduced the concentration of Na^+^ and Cl^−^ in the leaf and improved the water condition [4]; (3) Si increased the number and size of stomata [9], resulting in more CO_2_ entering leaf tissue [34], thus improving photosynthetic and transpiration efficiency, giving flexibility to photosynthetic function, and effectively improving the level of carbohydrate synthesis, which may be a reason for the increase in licorice growth and biomass. However, based on the results of the present study, this promoting effect was more obvious for *G. uralensis* under high-salt conditions. Notably, many factors affect licorice growth in changing environments. In particular, Si application to roots instead of leaves may directly or indirectly adjust the root configuration, physiology, and biomass [33] and then affect the soil environment through the rhizosphere. Soil affects roots and leaves through rhizosphere microorganisms or rhizosphere chemical processes. Therefore, future research needs to study the complete licorice plant and its soil environment to reveal the effect of foliar application of Si on licorice more comprehensively and systematically.

## Conclusion

Medium-salt and high-salt treatments had negative effects on the growth of *G. uralensis* and *G. inflata*; the inhibitory effect on *G. uralensis* was more pronounced. Foliar application of Si led to an increase in leaf water content, improvements in leaf morphological characteristics and conduction capacity of leaf vein vascular bundles, and increases in palisade tissue volume, chlorophyll content, and photosynthetic efficiency, and had a favorable effect on the growth and development of the two licorices under the tested salt concentrations. However, there were differences in the effects of Si in different salt environments based on plant species. In this study, the promotion effect of foliar Si application on *G. uralensis* was more significant under medium and high salt concentrations compared with that on *G. inflata*. Therefore, we suggest paying more attention to the effect of exogenous Si application on the growth of *G. uralensis* under salt stress in the future researches.

## Material and methods

### Experimental design

*Glycyrrhiza uralensis* and *G. inflata* were used as test materials. The pot experiment was conducted in an experimental field (44° 30′ N, 86° 06′ E) at the School of Life Sciences of Shihezi University, China, from the end of April to the beginning of November 2021. *G. uralensis* seeds were collected from Wenquan County (Xinjiang, China; 44° 58′ N, 80° 57′ E), and *G. inflata* seeds were collected from Korla (Xinjiang, China; 41° 69′ N, 86° 12′ E). Concentrated H_2_SO_4_ (98%) was used to break the dormancy of licorice seeds selected for full grains and consistent size, which were then disinfected with 0.1% HgCl_2_. Each pot (23.5 × 16 × 18 cm^3^) was filled with 5 kg of sandy loam. The basic characteristics of the soil were as follows: pH, 7.63; Si, 0.03 g kg^−1^; salt content, 2.91 g kg^−1^; available nitrogen, 42.55 mg kg^−1^; available phosphorus, 4.23 mg kg^−1^; available potassium, 82.51 mg kg^−1^; and soil organic matter, 5.83 g kg^−1^.

Each pot was seeded with eight seeds. When the seedlings grew two true leaves, four strong and disease-free seedlings with the same height and growth trend were retained in each pot. After the plants had grown for 30 d, salt and Si were applied simultaneously. The concentrations of salt and silicon were set according to the appropriate salt range of the two licorice [42] and the results of the previous preliminary experiment. Si was sprayed onto the leaves in the form of potassium silicate (K_2_SiO_3_) at a 3 mM concentration. The experiment adopted a completely randomized block design, with a total of six treatments: (1) 0 g kg^−1^ NaCl (CK), (2) 6 g kg^−1^ NaCl (medium-salt concentration, 6S), (3) 12 g kg^−1^ NaCl (high-salt concentration, 12S), (4) CK + Si, (5) 6S + Si and (6) 12S + Si; there were four replicates of each treatment. In order to eliminate the influence of K^+^ caused by K_2_SiO_3_ application on the test, and the effects of Cl^−^ on plants could be almost ignored when its concentration was low, 6 mM KCl was added to all treatment without Si [43]. During the experiment, 300 mL of water was used for watering daily. At the end of the experiment (100 d), the morphology, anatomical structure, Chl content, gas exchange parameters, and growth parameters of the two licorice leaves were measured.

### Determination of leaf morphological characteristics

Four licorice plants with the same growth were selected from each treatment; the number of leaves were measured using the counting method, and the area of mature leaves at the same position of each plant was measured using a leaf area analyzer LC-2400P. Fully developed leaves on the third or fourth branch from the plant apex were collected and weighed for the fresh weight (FW). The leaves were placed in distilled water for 12 h and removed to measure the turgid weight (TW). The sample was then placed in an oven at 75 °C for 12 h, removed, and measured for the dry weight (DW). The relative water content (RWC) of the leaf was calculated using the formula [44]:

RWC (%) = [(FW – DW) ⁄ (TW – DW)] × 100.

### Determination of leaf anatomy

Mature leaves at the same position (on the fourth branch from the plant apex) were selected, and placed in an FAA stationary solution (50% ethanol: glacial acetic acid: formaldehyde = 18:1:1). After dehydration in alcohol, clearing in xylene, embedding in wax, sectioning (8-μm thickness), staining with safranin-fast green, and sealing with Canadian gum, permanent slices of cross-sectioned leaves were made. Images were obtained with a polarized light microscope (BX51, Olympus, Germany), and photographs were taken with an Olympus DP70 micrograph system. Finally, the thickness of leaf, epidermis, main vein, palisade tissue, spongy tissue and the area of xylem, phloem, parenchyma, and vascular bundle of the main vein with Motic Images Advanced 3.2, and the ratio of the palisade to spongy tissue, tissue structure compactness, and porosity were calculated as follows:

Ratio of palisade to spongy tissue = [palisade tissue thickness]/ [spongy tissue thickness].

Tissue structure compactness = [palisade tissue thickness]/ [leaf thickness] × 100%

Tissue structure porosity = [spongy tissue thickness]/ [leaf thickness] × 100%

### Determination of photosynthetic gas exchange parameters

The net photosynthetic rate (*Pn*), stomatal conductance (*Gs*), transpiration rate (*Tr*), and intercellular CO_2_ concentration (*Ci*) were measured using a portable Li-6400 (Li-COR, Lincoln, USA) photosynthetic instrument between 10:00 and 12:00 a.m., with a light intensity of 1000 μmol∙m^−2^ s^−1^, leaf chamber temperature of 28 °C, and CO_2_ concentration of 400 μmol∙mol^−1^ and Each treatment was repeated four times.

### Determination of Chl content

After removing the veins, fresh leaves were cut into 0.2 g pieces, to which 10 mL acetone (80%, v/v) was added. After being placed in the dark for 24 h (the fragments of the leaves became colorless), the absorbance values of the extracted solution at 470, 646, and 663 nm were determined using a spectrophotometer (UV-2100). The Chl a, Chl b, and Car contents were calculated as follows [45]: Chl a = 12.21A_663_-2.81A_646_; Chl b = 20.13A_646_-5.03A_663_; Car = (1000A_470_ -3.27Chl a-104Chl b)/ 229; total Chl content = Chl a + Chl b.

### Determination of growth parameters

Plant heights were measured using tape, and the total dry weight was recorded. The stems, leaves, and roots of the plants were washed, placed in a drying oven, dried at 75 °C for 48 h, and then weighed.

### Statistical analysis

The data were analyzed using the SPSS statistical package (version 20.0; IBM, Armonk, New York, USA). The error bars offer the average value of four replicates. Data were analyzed at the significance level of *P* < 0.05 using the ANOVA two-way and Duncan’s multiple range tests.

## Supplementary Information

**Additional file 1: Table S1**. Two factor ANOVA (Si and NaCl treatment) for all parameters studied of licorice significance values.

## Data Availability

The datasets used and/or analysed during the current study available from the corresponding author on reasonable request.

## Abbreviations

Car: Carotene
Chl: Chlorophyll
*Ci*: Intercellular CO_2_ concentration
*Pn*: Net photosynthetic rate
*Gs*: Stomatal conductance
*Tr*: Transpiration rate
DW: Dry weight
FW: Fresh weight
RWC: Relative water content
TW: Turgid weight
