# Supplementary material for: Effects of silicon application on leaf structure and physiological characteristics of Glycyrrhiza uralensis Fisch. and Glycyrrhiza inflata Bat. under salt treatment
Source: BMC Plant Biol. 2022 Aug 4;22:390. doi: 10.1186/s12870-022-03783-7 (PMC9351143; doi:10.1186/s12870-022-03783-7)
Supplement: Supplementary file 1 — Additional file 1: Table S1. Two factor ANOVA (Si and NaCl treatment) for all parameters studied of licorice significance values. [file 12870_2022_3783_MOESM1_ESM.docx]

| Table S1 Two factor ANOVA (Si and NaCl treatment) for all parameters studied of licorice significance values | | | | | | |
| --- | --- | --- | --- | --- | --- | --- |
| Parameters | *Glycyrrhiza uralensis* | | | *Glycyrrhiza inflata* | | |
|  | Si | NaCl | Si × NaCl | Si | NaCl | Si × NaCl |
|  | *F* | *F* | *F* | *F* | *F* | *F* |
| Leaf area | 14.95^**^ | 17.40^**^ | 0.61 | 4.59^*^ | 21.86^**^ | 0.56 |
| Leaf number | 106.05^**^ | 420.06^**^ | 0.42 | 49.22^**^ | 245.31^**^ | 0.06 |
| Relative water content | 0.24 | 9.10^**^ | 1.48 | 0.75 | 14.39^**^ | 0.12 |
| Xylem area | 285.95^**^ | 336.91^**^ | 38.84^**^ | 83.98^**^ | 284.18^**^ | 41.14^**^ |
| Phloem area | 2225.76^**^ | 3227.74^**^ | 224.94^**^ | 168.32^**^ | 962.83^**^ | 8.02^**^ |
| Vascular bundle area | 1474.16^**^ | 3036.17^**^ | 268.84^**^ | 671.90^**^ | 2235.86^**^ | 121.14^**^ |
| Parenchyma area | 67.85^**^ | 81.82^**^ | 7.01^**^ | 2.35 | 32.31^**^ | 14.85^**^ |
| Main vein thickness | 60.79^**^ | 75.51^**^ | 4.25^*^ | 1.77 | 7.34^**^ | 1.74 |
| Leaf thickness | 0.001 | 30.08^**^ | 3.02 | 12.99^**^ | 110.33^**^ | 40.24^**^ |
| Palisade tissue thickness | 3.75 | 24.12^**^ | 1.74 | 1.11 | 36.24^**^ | 12.66^**^ |
| Sponge tissue thickness | 18.22^**^ | 27.61^**^ | 2.57 | 67.30^**^ | 96.46^**^ | 45.78^**^ |
| Ratio of palisade to sponge | 19.50^**^ | 3.46 | 0.81 | 35.71^**^ | 36.06^**^ | 3.29 |
| Epidermal thickness | 11.74^**^ | 1.61 | 3.28 | 22.88^**^ | 52.35^**^ | 2.70 |
| Tissue structure compactness | 2.72 | 0.52 | 1.26 | 12.72^**^ | 14.84^**^ | 0.28 |
| Tissue structure porosity | 16.61^**^ | 4.91^*^ | 0.28 | 43.71^**^ | 36.20^**^ | 14.49^**^ |
| Chlorophyll a | 16.24^**^ | 177.77^**^ | 1.87 | 7.89^*^ | 50.30^**^ | 1.76 |
| Chlorophyll b | 14.83^**^ | 39.36^**^ | 2.44 | 0.98 | 37.06^**^ | 0.37 |
| Total chlorophyll | 23.77^**^ | 200.39^**^ | 1.75 | 7.75^*^ | 61.05^**^ | 1.77 |
| Carotenoid content | 25.79^**^ | 206.91^**^ | 1.90 | 17.73^**^ | 119.61^**^ | 2.70 |
| Net photosynthetic rate | 124.03^**^ | 606.55^**^ | 18.01^**^ | 16.61^**^ | 737.96^**^ | 0.56 |
| Transpiration rate | 35.56^**^ | 498.57^**^ | 25.43^**^ | 12.77^**^ | 351.97^**^ | 4.35^*^ |
| Intercellular CO_2_ concentration | 1.56 | 87.21^**^ | 44.73^**^ | 44.72^**^ | 159.65^**^ | 3.55^*^ |
| Stomatal conductance | 7.18^**^ | 318.69^**^ | 2.69 | 28.86^**^ | 341.57^**^ | 1.69 |
| Plant height | 182.79^**^ | 312.29^**^ | 6.01^*^ | 79.91^**^ | 179.34^**^ | 10.50^**^ |
| Dry weight | 44.67^**^ | 851.84^**^ | 1.89 | 44.48 | 1113.30^**^ | 1.53 |
| * indicate significant correlation at the 0.05 level, ** indicate significant correlation at the 0.01 level. | | | | | | |
